# Supplementary material for: Are carbon emissions trading and green financial instruments synergistic? -Comprehensive quantitative research based on content analysis
Source: PLoS One. 2024 Mar 7;19(3):e0298601. doi: 10.1371/journal.pone.0298601 (PMC10919601; doi:10.1371/journal.pone.0298601)
Supplement: S1 Data — (ZIP) [file pone.0298601.s001.zip › Data/地级市Z_F工作报告.zip!/║╙▒▒/╒┼╝╥┐┌╩╨/└┤╫╘╣1⁄2╓┌║┼í╛┬φ┐╦╩2╛▌═°í┐_2016.docx]

各位代表：

现在，我代表张家口市人民政府向大会作工作报告，请各位代表连同《张家口市国民经济和社会发展第十三个五年规划纲要(草案)》一并审议，并请市政协委员和列席会议的同志提出意见。

一、“十二五”时期经济社会发展回顾

“十二五”时期是我市经济持续健康发展、社会事业全面进步、人民生活水平不断提高的五年。在省委、省政府和市委的坚强领导下，全市上下坚持“稳中求进”工作主基调，努力破解改革发展难题，凝心聚力，拼搏奋进，圆满完成“十二五”规划主要目标任务，为全面建成小康社会奠定了坚实基础。总结五年来的工作和成绩，主要有以下九个方面：

致力稳增长、求发展，综合经济实力不断增强。全市生产总值由2010年的966.4亿元增加到2015年的1363.5亿元、五年年均增长8.1%，人均生产总值由22517元增加到30808元、年均增长7.4%，全部财政收入由144.8亿元增加到230.7亿元、年均增长9.8%，一般公共预算收入由62.4亿元增加到133亿元、年均增长16.3%，一般公共预算支出由181.7亿元增加到391.3亿元，年均增长16.6%。固定资产投资达到1554.2亿元、年均增长11.9%，规模以上工业增加值达到404.7亿元、年均增长9.7%，社会消费品零售总额达到615.6亿元、年均增长 14%。实际利用外资完成13.7亿美元、是“十一五”的4倍，外贸进出口总额达到6.7亿美元、年均增长18.6%政府工作报告。

坚持讲大局、聚合力，申办冬奥一举获得成功。2013年10月，党中央、国务院批准京张联合申办2022年冬奥会后，我市始终坚持“一切工作围绕和服从于申奥”大局，高水平实施了迎宾廊道和赛场核心区周边10.5万亩造林绿化，下大力整治了沿线环境，全方位营造了全民参与申奥的浓厚氛围。配合北京高质量完成了调查问卷、视频质询、申办报告和洛桑、吉隆坡两次陈述，通过了国际体育单项组织对5个比赛场地的认证，圆满完成了迎接国际奥委会评估团实地考察工作。2015年7月31日一举赢得2022年冬奥会举办权，创造了“一次申办、一次成功”的奇迹。申奥成功，将对我市长远发展产生重大影响。

着力调结构、兴产业，转型升级取得积极进展。大力化解高耗过剩产能，努力改造提升传统产业，加快培育壮大战略性新兴产业和现代服务业。累计压减炼钢产能384万吨、炼铁产能366万吨、水泥产能458.6万吨，矿山、钢铁、化工、水泥四大高耗能产业占规模以上工业的比重由2010年的48.9%下降到了36%。全市单位GDP能耗提前一年完成省下达任务。累计完成技改投资1324.2亿元，被工信部认定为全国首批工业绿色转型发展试点城市。新兴产业发展迅速，建立了总规模47.8亿元的产业基金，高端制造、新型能源、电子信息等高新技术产业实现增加值比2010年增长1.5倍，年均增长19.8%。全市高新技术企业由2010年的12家增加到40家，科技型中小企业由2013年开始统计时的380家增加到760家。挂牌上市企业达到18家，其中长城乳业、时代橡胶成为首批新三板挂牌企业。服务业发展提速，对经济增长的贡献率高于二产17.7个百分点。旅游业收入年均增长38.6%，康保县省级电子商务试点建成了“一县多品多平台”体系。农业现代化步伐加快，特色产业持续壮大，市级以上龙头企业达到425家、其中国家级3家。畜牧、蔬菜、果品、马铃薯四大产业占农业总产值比重达到78.2%。三次产业结构由15.8∶43∶41.2调整为17.9∶40∶42.1。

立足促协同、做示范，重点领域实现重大突破。京张两地往来日益紧密，多方面融合加速推进。张北云计算产业园、怀来航空航天产业基地等一批转移平台初步建成，福田泵车、智慧能源等一批重大项目开工建设。社会事业协同发展迈出实质性步伐，北京天坛医院张家口脑科中心、北京中医院合作医院挂牌成立。市职教中心入选北京现代服务业职业教育集团常务理事单位。五年共引进北京资金1496亿元，其中2015年引进338.1亿元。国务院批准张家口可再生能源示范区建设，与国网节能、新奥集团、特变电工、金风科技等多家企业在能源互联网、能源装备制造等方面开始了探索实践。2015年风电装机容量、并网发电分别达到 736万千瓦和695万千瓦，光电装机并网达到70万千瓦，位列全国地级市前列。新能源产业增加值占规模以上工业增加值的比重达到了10.2%。

突出抓项目、建园区，经济发展模式日益成熟。五年共实施重点项目910项，完成投资2885.3亿元。实施了沃尔沃发动机及整车制造、旗帜乳业、风光储输、蔚县电厂、云顶密苑滑雪、太舞滑雪小镇、盛唐葡萄酒、乾信牧业等一大批重大产业项目。沃尔沃发动机累计下线10.62万台，缸体、缸盖生产线已投入运行，整车项目已完成大部分主体工程，生产资质获工信部批准，今年10月份张家口生产的第一台汽车将正式下线。园区建设快速推进，全市工业园区发展到23 个，省级园区增加到16个，建成区面积达到56.9平方公里。特别是通过“股权投资+科技项目+专业园区”的模式，与上海张江合作建设了张江张家口高新技术产业园，建成了21万平方米的高科新城科研生产基地。阿里巴巴等8个大数据项目落户张北云计算产业园。西山产业集聚区被工信部确定为国家新型工业化产业示范基地。怀安工业园成为全国首批国家应急产业示范基地。综合性现代农业园区达到15个，塞北成功创建国家级现代农业示范区。

努力多绿化、治污染，生态环境持续改善。五年累计实施各类林业生态建设工程593万亩，森林覆盖率达到37.05%，较2010年增加了5.4个百分点。建成国家级自然保护区2个、森林公园2个，新增国家湿地公园试点4处。在全省率先实现国家生态功能区补助范围市域全覆盖，累计争取资金36.2亿元。崇礼成为北方地区唯一的全国首批碳汇城市。大力推行节水农业，五年新增及改善节水灌溉面积136.58万亩。以“零容忍”的态度防治大气污染，重点对煤烟污染和企业排放问题进行整治，对17台燃煤发电机组实施了超低排放改造，淘汰和改造燃煤锅炉1297台，取缔高速及国省干道两侧煤栈821家，拆除粘土实心砖瓦窑140座，主城区搬迁污染企业9家，淘汰黄标车10.89万辆，投入使用新能源车1056辆。大气环境质量连续三年在京津冀地区保持最好水平。成功创建国家园林城市和国家森林城市，入围国家第一批生态文明先行示范区、生态保护和建设示范区。

注重夯基础、补短板，城乡社会面貌显著提升。张唐铁路竣工通车，张呼、张大客专开工建设。张涿高速、张石三期、张承二期、北绕城高速竣工通车，京新三期、二秦高速开工建设，高速公路通车总里程达到926.8公里，位居全省前列。24条段地方道路升级为国省干线公路。宁远机场通航并开通五条航线，2015年旅客吞吐量达到20.8万人次。张家口海关开关运营良好。我市行政区划调整方案获得国务院批准，主城区面积由868.96平方公里扩展到 6377.81平方公里，城市承载功能进一步增强。主城区开工建设了集中供热、城市道路及图书馆、博物馆等一批市政基础设施和公共服务设施。阳原县、经开区、崇礼、万全初步实现了城乡环卫一体化。张北、怀来创建为国家园林县城，涿鹿等7个县成为省级园林县城。全市常住人口城镇化率达到51.3%，比 2010年提高了6.13个百分点。美丽乡村建设扎实推进，改造提升693个重点村，农村生产生活条件明显改善。

着眼转职能、增活力，各项改革深入推进。市本级精简审批事项达到了442项，精简比例为71%。全部取消了非行政许可审批事项。市级公共资源交易中心平台整合工作全面完成。“三证合一、一照一码”登记制度全面实施，“四级平台、三个代办”工作全面展开。大众创业、万众创新活力涌现，市场主体达到了18 万户，比2010年增加7.17万户，万人拥有市场主体数量由250户增长到408户。建成高层次人才创新创业园4家、大创园及创业孵化基地19家。政府机构改革顺利完成。农村改革稳步推进，农村土地承包经营权确权登记完成率达到60.6%，土地流转299万亩，流转率达到29%。供销社综合改革提升工作取得成效。切实加强政府系统党风廉政建设，全面推进依法行政，政府系统工作作风进一步转变。自觉接受人大、政协的监督，认真听取各民主党派、工商联和无党派人士意见建议，人大代表建议、政协委员提案办复率100%。

围绕办实事、保和谐，民生和社会事业稳步发展。五年来全市财政用于民生支出达到1185.1亿元，占全部财政支出的78.1%，年均增长19.4%。城乡居民人均可支配收入分别达到23841元和8341元，年均分别增长10.2%和15.2%。城镇累计新增就业26.2万人。圆满完成国家“全民参保登记”试点工作。城镇居民医保和新农合财政补贴标准比2010年提高216.7%，达到380元，城镇、农村低保年保障标准比2010年分别提高66%和 73%，分别达到4770元和2250元。企业退休职工养老金连续11年上调，累计提高110%，达到1976元。农村互助幸福院、城市社区居家养老服务中心覆盖率分别达到65%和100%。累计建设各类保障性住房9.33万套，分配入住4.49万套，完成农村危房改造15.4万户。加大产业扶贫、精准扶贫力度，80万贫困人口实现稳定脱贫。新改扩建农村标准幼儿园300多所，主城区7所中小学完成新建或扩建，职业教育和特殊教育达到全国先进水平，市职教中心被誉为“全国职业教育的一面旗帜”，张家口学院完成了改制升本工作。医药卫生改革成效明显，覆盖城乡的公共卫生和医疗服务体系基本建立。一批具有张家口地域特色的文化精品力作不断推出，打造了中华三祖、阳原泥河湾等文化品牌。实施了察哈尔都统署、大境门、张北元中都等遗址保护项目;5 个县的16个村入选首批国家传统古村落保护名录。举办了第四届环北京职业公路自行车赛等大型赛事和群众性健身活动。我市残疾人运动员赵帅在仁川亚洲残运会上获得两枚金牌。张杂谷成为我市拥有自主知识产权并具有国际领先水平的科技成果，赵治海荣获“何梁何利基金奖”。建成了2个国家级高新技术产业化基地、1 个国家级国际合作基地。全市共获得国家、省级各类科技奖励82项。启动开展了国家食品安全试点和安全发展城市创建工作，和谐稳定发展局面进一步巩固。国防动员、民族宗教、新闻广电出版、史志档案、气象地震、援藏援疆、妇女儿童、老龄、残疾人等方方面面的工作都取得了新的成绩。

2015年是“十二五”规划的收官之年，也是张家口历史上非常不平凡的一年，大事多、机遇多、挑战多、困难多。一年来，面对申奥的重大任务，我们上下齐心，全力以赴，全面夺取最后的成功;面对经济下行压力，我们沉着应对，多措并举，发展换档不失速;面对三大历史机遇，我们精心谋划，积极作为，科学理清了城市定位、发展理念、主导产业和主攻方向。整体经济社会发展呈现出稳中有进、稳中向好的态势，全市生产总值同比增长5.8%，一般公共预算收入增长5.73%(可比增长9%)，固定资产投资增长10.9%，规模以上工业增加值增长4.2%，社会消费品零售总额增长9.5%，实际利用外资增长2.3%，外贸进出口总额增长28.4%，城乡居民人均可支配收入分别增长10.1%和11.8%。

各位代表!这些成绩的取得，凝聚着全市上下的辛劳与汗水，凝聚着社会各界的关心与支持。在此，我代表市人民政府，向全市人民，向人大代表、政协委员，向各民主党派、工商联、无党派人士和人民团体，向驻张人民解放军、武警官兵、政法干警，向所有关心、支持张家口建设和发展的各级领导、离退休老干部、各界人士和海内外朋友表示衷心的感谢，并致以崇高的敬意!

在总结成绩的同时，我们也要看到，在经济发展和社会进步方面，还存在诸多问题和困难。一是经济欠发达的现状尚未根本改变。2015年全市生产总值在全省排第8位，仅占全省总量的4.6%。人均生产总值在全省排第8位，为全省的77%。一般公共预算收入在全省排第7位，人均水平为全省的83.8%。城乡居民人均可支配收入在全省分别排第7位和第10位，分别为全省的91.2%和75.5%。二是城乡建设水平仍较落后。老城区道路、绿化、文体设施、停车场地建设等欠账较多，棚户区、城中村改造任务较重，城乡脏乱差问题突出。市民素质有待提高，城市文明程度还需提升。农村基础设施和公共设施建设严重滞后，村庄面貌总体较差。三是脱贫攻坚和社会保障压力仍然较大。全市有45万农村贫困人口和10万城市低收入群体，医疗教育等基本公共服务供给不足，保障和改善民生任务艰巨。城乡缺水问题日益严重，对水源涵养功能区和生态环境支撑区建设带来巨大挑战。四是一些单位和部分干部担当意识和服务意识不强，行政效率不高，营商环境还需要进一步优化。另外，需要报告的是，受宏观经济形势下滑、税收政策调整、环保限制愈发严格等因素的影响，2015年地区生产总值、固定资产投资、规模以上工业增加值、社会消费品零售总额、实际利用外资5项主要经济指标没能完成年初确定的目标任务。对于这些问题，在今后工作中，我们将以切实有效的措施认真加以解决。二、“十三五”时期的主要目标和任务

“十三五”时期，是全面建成小康社会的决胜时期，是我市三大机遇相互叠加、效应集中释放、机遇优势转化为现实生产力的关键时期，是推动转型升级、实现绿色崛起最为宝贵的时期。我们必须创新发展理念，用足用好机遇，顺势而为，乘势而上，夙兴夜寐，激情工作，推动跨越发展、绿色崛起。

“十三五”时期我市经济社会发展的指导思想是：全面贯彻落实党的十八大和十八届三中、四中、五中全会精神，以邓小平理论、“三个代表”重要思想、科学发展观和习近平总书记系列重要讲话为指导，深入贯彻中央和省委的决策部署，牢固树立创新、协调、绿色、开放、共享的发展理念，抢抓京津冀协同发展、京张携手举办冬奥会、建设国家可再生能源示范区三大机遇，坚持“转型升级、跨越发展、绿色崛起”主基调，坚持“解放思想、担当实干”总要求，坚持“多种树、兴水利、促转型”，坚守发展、生态、民生三条底线，大力培育大生态、大旅游、大数据、大健康和新能源、新技术、高端制造“四大两新一高”主导产业，着力打造水源涵养功能区、绿色产业聚集区、可再生能源示范区、国际休闲运动旅游城市和奥运名城，统筹做好调结构、促改革、优环境、惠民生各项工作，着力提升各级干部执行力，确保如期全面建成小康社会。

“十三五”时期我市经济社会发展的主要目标是：“两个进位、两个翻番、四个全部、一个全面建成”。“两个进位”，就是经济总量实现进位，进入全省中游以上行列，生产总值年均增长8%左右;空气质量综合指数实现进位，达到世卫组织第二阶段标准。“两个翻番”，就是全市生产总值和城乡居民收入均比2010年翻一番。“四个全部”，就是贫困县全部摘帽，贫困村全部出列，贫困人口全部脱贫，美丽乡村全部覆盖。“一个全面建成”，就是2020年如期全面建成小康社会。

实现以上目标，今后五年我们主要从四个方面、抓好八项重点工作。

(一)完成“三大历史任务”

未来五年，筹办冬奥会、推进生态建设、打赢脱贫攻坚战，是我们要承担的三大历史任务，必须不折不扣地全面推进。

—全力以赴抓好冬奥会筹办和奥运经济发展。我们将认真贯彻落实习近平总书记“绿色办奥、共享办奥、开放办奥、廉洁办奥”的总要求，坚持世界眼光、国际标准、奥运思维，实行6年倒计时工作制，全面完成国际奥委会规定的任务，为全世界展现一届“精彩、非凡、卓越”的冬奥会。通过“奥运+”计划，推动全市经济社会快速发展，加快打造国际化、现代化城市。高水平做好场馆和基础设施的规划建设。到2020年完成所有场馆建设任务，并具备测试赛条件。建成一批铁路、公路和通用机场项目。把“以运动员为中心、可持续发展、节俭办赛”三大理念贯穿于筹办和举办全过程，深度挖掘和融入“长城、泥河湾、三祖、中国年” 等历史文化，把冬奥会办出民族特色，达到世界一流水平。充分利用社会资源办奥运，按照国际通行的办法进行项目管理和监督审计，打造“像冰雪一样纯洁干净” 的冬奥工程。全面推广冰雪运动。加强冰雪场地建设，组织系列冰雪季活动，在全市中小学和广大市民中推广冰雪运动。培养张家口冰雪专业运动队伍，举办各类高层次、高水平的体育比赛，积累办赛经验，提高运营水平，进一步扩大“崇礼滑雪”的世界影响。围绕举办冬奥会的需求，充分利用好我市职业教育资源，有计划、有针对性地培养外语、滑雪、教练、医疗等各类专业人才和志愿者。大力发展奥运经济。打造以崇礼为核心、周边县区为补充的冰雪大区，把我市建成世界冰雪运动基地。借助冰雪旅游和体育运动，加快建设一批各具特色的冰雪风情小镇，培育壮大与之配套的现代服务业。选择合适的园区，集中引进国内外高端冰雪装备、设备制造企业，打造冰雪旅游、装备制造、产业服务为一体的冰雪产业链。以奥运为媒介，进一步扩大对外开放，积极推进与日韩、欧美等国家和地区的交流合作，在引进国外资金、技术、项目上实现较大突破。

—坚持不懈推进生态建设。立足“京津冀水源涵养功能区”的定位和冬奥会的特殊要求，大幅提升生态环境质量。加快国土绿化。对全市所有道路、村庄、沿京、沿坝等区域全面绿化，每年新造林110万亩以上，确保到2022年森林覆盖率达到50%以上。坚持“绿”和“富”相结合，提高经济林在造林中的比重。推进全域生态系统修复，2020年基本关闭取缔对生态环境破坏大的煤矿、铁矿及露天开采矿山。大力兴建水利。坚持农业节水、地表蓄水、域外调水相结合，五年之内实现节水灌溉全覆盖。加快地下水超采综合治理，谋划推进域外调水等重大水利工程，切实解决水资源严重短缺的问题。推进湿地修复，搞好湿地公园保护，加强湿地水源涵养。改善大气质量。以世界卫生组织第二阶段标准为目标，综合实施“减煤、降耗、治企、抑尘、控车”等大气污染防治措施，引导大众参与，严格环境执法。重点推进能源结构调整和新能源替代，到2020年能源替代率达到或接近三分之一，京张奥运绿色智慧走廊和低碳奥运专区基本建成。

—全面完成脱贫攻坚和美丽乡村建设任务。坚持扶贫开发与全面建成小康同步推进、与美丽乡村建设紧密结合，按照“六个精准”的要求，把产业发展作为精准扶贫的核心，更加有效地推进“六个一批”。广泛动员全社会力量参与扶贫事业。到2018年底，实现全市贫困县全部摘帽，贫困村全部出列，贫困人口全部脱贫。大力推进美丽乡村建设，坚持与特色农业、农家旅游等相结合，突出地域特色，因地制宜、分类施策，实施好新能源替代、民居改造等“十二个专项行动”，重点采取精品示范、综合整治、文化旅游开发、中心村建设四种模式，着力建设生态美、产业美、环境美、精神美的“四美新农村”。

(二)全力办好“两件大事”

未来五年，协同发展和可再生能源示范区建设是推动我市跨越发展的强大引擎，必须全力以赴地高效推进。

—全力对接协同发展重大战略。加速交通互联互通。打通京张铁路快速通道、高速公路“对接路、绕京线”和国省干道“瓶颈路”，建设首都1小时通勤圈，畅通进京通道，打造网格状、全覆盖的现代综合交通架构。加快打造承接转移平台。以承接教育、医疗、产业为重点，支持各县区因地制宜建设不同层次、各具特色的协同发展基地，积极跑办和争取北京的资源向我市转移。推进与北京签订的卫生、产业等合作协议，建立教育、医疗合作机制，共同实施奥运医疗保障项目，推进两地公共事业融合发展。加强生态环境共建。与北京共同构建生态协作推进机制和横向补偿机制，推动京冀水源林项目由5个县逐步扩大至全域。联合建立生态投资专项基金，共同实施生态修复、饮用水源地保护等生态环境工程。深化与北京的科技合作。与北京科研院所、高等院校建立更加紧密、更加直接的联系，共建科技成果发布平台、产业技术创新联盟、技术交易市场，争取一批国家重点实验室、工程技术中心、重大科技基础设施等向我市布局，建成北京科技成果转化的重要基地。

—全力建设可再生能源示范区。围绕发、储、输、用四大环节，组织实施规模化有序开发、大容量储能应用、智能化输电通道建设、多元化应用示范四大工程，推进低碳奥运专区、科技创业城、综合商务区、高端装备制造聚集区和农村可再生能源生态示范区五大功能区建设，2020年可再生能源发电装机规模达到 1280万千瓦，新增装机580万千瓦，消费量占终端能源消费总量30%以上，基本建成全国首个低碳专区。积极争取政策，强力推动体制改革、商业模式和先进技术三大创新取得突破。到2020年崇礼区乡村全部使用泛能网，实现可再生能源的全覆盖，建成国际领先的低碳专区，在国内可再生能源创新应用等方面先行先试。

(三)努力实现“两个新突破”

未来五年，产业结构优化和新型城镇化是我市经济社会持续发展需要着力突破的重点领域，必须扎扎实实地不懈推进。

—推动转型升级实现新突破。大力培育“四大两新一高”主导产业。加快新兴产业发展，构建新的产业结构。大生态，重点发展规模化、集约化、品牌化的生态林业、现代农业，促进产业发展生态化、生态建设产业化。大旅游，引进大企业、采取大投入、进行大开发、打造大品牌，规划、整合全市旅游资源，大力提高旅游业发展的层次和水平，形成全区域、全要素、全时段、全品种的旅游形态，打造国际休闲运动旅游城市和冬奥名城。大数据，以张北云计算产业园为中心，进一步向坝上其它县区延伸，推进下花园智慧环境产业园等项目，推动产业链条逐步从基础硬件、系统平台向应用增值等环节延伸，以大数据带动信息产业发展，加快打造“中国数坝”。大健康，加强与北京医疗机构的合作，发展医疗+养老、保健、康复、制造的新型业态，并在进口医药设备、中外合资办医等方面争取国家特殊政策支持，打造高端健康产业的集聚区。新能源，以国家可再生能源示范区建设为载体，通过规模化、多元化、创新式、链条式开发应用，建设全形式、全过程、高技术、高聚集、智慧化、可复制的示范基地。新技术，积极承接科研成果孵化转化，以高新技术产业园为载体，引进培育一批生物医药、电子信息、物联网、新材料等领域的科技型、创新型企业，打造高新技术产业集群。高端制造，以智能制造为核心，重点发展汽车发动机、整车和零部件配套、新能源设备、应急装备、特种和专用汽车、航空航天设备、智能机器人等高附加值产业，逐步培育形成先进制造业集群。大力提升传统企业发展水平。坚持工业化和信息化深度融合，用好国家工业绿色转型发展试点城市政策机遇，加大技术改造力度，改进生产装备、工艺水平，提升产品质量，逐步向价值链高端拓展延伸。

—推动城乡统筹发展实现新突破。加快城市现代化。以城市区划调整为契机，进一步优化完善城市建设规划、空间布局和功能分区，统筹城市规划建设和管理，切实转变城市发展方式。旧城区推进以完善配套设施为重点的改造提升，新城区抓好以展示现代化城市魅力为重点的规划建设。严格保护天际线和山际线，建设山、水、城相映衬的特色城市。大力发展楼宇经济、总部经济和商务集聚区等城市经济，扩大现代服务业比重。推进智慧城市建设，加快立法进程，通过教育和强制两种手段，形成城市规矩，打造城市品牌，提高城市管理水平。深入开展“迎奥运、讲文明、树新风”为主题的市民素质提升系列活动，创建全国文明城市、国家卫生城市。大力推进强县进程。树立产城教融合的理念，完善公共设施，加快扩容提质，吸引人口向县城集中，加速大县城建设进程。按照区域化、差异化、特色化的思路，形成“一带两翼”的县域经济发展格局。进一步转变农业发展方式，加强现代农业园区建设，推行标准化生产，健全社会化服务、农业科技推广、农产品质量安全监管体系，推广有机农畜产品认证工作，促进高效、绿色、生态、品牌、安全农业发展。建设布局集中、产业集聚、配套完善、特色鲜明的专业化产业园区，壮大提升县域产业，打造独具特色的板块经济，推动县域经济实现增比进位。“十三五”末，全市一半以上的县综合经济实力在全省实现位次前移。怀来、张北、蔚县建成副中心城市。

(四)扎实做好“一个保障”

未来五年，改善民生是我市跨越发展、绿色崛起的出发点和落脚点，必须始终如一地持续推进。

实现更高质量就业。推动大众创业、万众创新，实现更加充分、更加公平和更高质量的就业。完善创业扶持政策，营造宽松便捷的就业环境，放宽创业准入领域和条件，落实减税降费政策，促进自主创业，以创业带动就业。千方百计增加居民收入。健全工资正常增长机制，完善最低工资和工资支付保障制度，推行企业工资集体协商制度。着力提高中低收入群体收入水平。增加农民土地增值收益。健全社会保障体系。建立统一的城乡居民基本医疗保险制度，完善企业职工养老保险转移接续政策，推进与京津医疗保险转移接续无障碍通道建设。认真落实低保、优抚、救助政策，保障困难群众基本生活。努力发展教育文化事业。进一步推动各级各类教育协调发展，均衡配置城乡教育资源，统一城乡义务教育经费保障机制，促进教育公平。繁荣文化事业，发展文化产业，树立“大好河山张家口”特色文化品牌，加强历史文化遗产保护。切实提高全民健康水平。深入实施“健康张家口”行动计划，促进公共卫生服务均等化。加强重大疾病预防，大力发展中医药事业，全面提高医疗服务水平。逐步建立基层首诊、分级诊疗、双向转诊的医疗服务模式。认真落实好“全面两孩”政策，推进医养结合，积极应对人口老龄化。加快体育设施建设，发展体育事业和体育产业，提升人民健康素质。建立健全公共安全体系。完善食品药品监督管理体系，加快电子监管追溯制度、质量标识制度和食品药品诚信制度建设。依法打击制售假冒伪劣产品行为。进一步健全安全生产管理体制，强化隐患排查治理、风险预防控制和社会共治体系建设，遏制重特大安全事故发生。着力构建常态化、立体化社会防控体系，健全与首都综合执法联动机制。依法严密防范和惩治各类违法犯罪活动，加强“反恐”工作，促进社会和谐稳定。三、2016年主要工作

今年是“十三五”的开局之年，也是筹办冬奥会、京津冀协同发展、可再生能源示范区建设开展实质性工作的关键之年，做好今年的政府工作至关重要。我们将认真落实中央和全省系列工作会议精神，全面贯彻五大发展理念，适应和引领经济发展新常态，坚持“转型升级、跨越发展、绿色崛起”主基调，大力推进供给侧结构性改革，去产能、去库存、去杠杆、降成本、补短板，加快发展动能转换，提升发展质量效益。全年主要预期目标是：全市生产总值增长7.5%左右，一般公共预算收入可比增长8%，财政收入占生产总值比重提高0.5个百分点，固定资产投资增长13%，社会消费品零售总额增长10%，实际利用外资增长10%，外贸进出口总额增长8%，单位GDP能耗下降4%，PM2.5浓度控制在34微克/立方米以内，化学需氧量、氨氮、二氧化硫、氮氧化物排放量分别比上年削减 0.5%、0.3%、1.5%、1.8%，城乡居民可支配收入分别增长9%和9.5%，居民消费价格指数涨幅控制在3%以内，城镇登记失业率控制在 4.5%以内。

为实现今年目标任务，我们将通过“抓机遇、促转型、提水平”，统筹推进各项重点工作。

(一)千方百计抓住机遇，努力把优势转化为现实生产力。以时不我待的紧迫感，将一个个机遇落实为一项项工作，全面推进各个领域、各个层面、各项工作实现跨越提升。

1.集中力量，全面启动冬奥会各项筹办工作。按照国际奥委会的要求和北京冬奥组委的统一部署，扎实、有序、高效做好各项筹办工作。做好系列规划编制并启动有关建设工程。配合北京搞好总体规划、场馆规划，积极主动完成我市“1+1+X”系统规划，有序推进赛区道路、用水等八大配套工程，加快延崇、沽怀、京北、张沽线等外围道路及京张高铁崇礼线的前期工作，开工建设万龙至转枝莲等场区道路。确保完成比赛场馆及重大基础设施的土地收储工作。以县区为主体，市级统筹，精心组织，强力推进，依法征收与全面细致做好群众工作相结合，保护合法与打击非法相结合，严格落实工作方案，确保土地征收过程平稳顺利，逐步打造规范化、法制化、和谐稳定的土地征收模式。年底前完成赛场核心区3个村和京张高铁张家口段的土地征收任务。推进赛场核心区及周边村庄改造。对赛场核心区及崇礼城区通往雪场和草原天路沿线的23个村庄，实行“四个一”建设模式，高标准建设美丽乡村，实现村庄景区化、设施旅馆化、生活低碳化、旅游智慧化。年初规划动工，年内基本建成并具备接待能力，在全市率先建成美丽乡村的标杆村、样板村。大力推广冰雪运动。引进世界顶级规划单位，对市域内的雪场资源进行规划，加快推进崇礼翠云山、四季小镇和赤城新雪国等滑雪场建设，不断扩大冰雪场地规模。建设张家口学院、市职教中心、经开区、崇礼区4处滑冰场和沽源滑冰馆。配合省体育局建设省级冰雪训练基地，促成国家体育总局建设国家队滑雪训练基地，打造中国冰雪运动中心。精心举办好“大好河山·激情张家口”冰雪季、国际儿童滑雪节、京张冰雪挑战赛等群众性冰雪活动，以及国际雪联远东杯滑雪赛等系列国际、国内专业赛事。推动与奥地利基茨比尔、日本长野、加拿大温哥华的合作，组织好中日韩冬奥交流合作等活动，学习借鉴赛事运营管理先进方法，安排我市滑雪运动员参加国内集训和国外培训活动。

2.主动出击，推动京张合作取得更好成果。全面贯彻国家规划纲要、河北省实施意见和我市推进措施，确保一批合作项目落地见效。大力承接非首都核心功能转移。在环京及主城区等地，高起点、高标准建设一批非首都核心功能承接“微中心”和教育、医疗、产业园区，谋划推进怀来、涿鹿的科教园区建设。吸引北京中高职及本科院校来我市建院设校、发展产业。巩固扩大京张医疗卫生合作成果，开展进修培训、协作办医，着力推动崇礼医院与北京积水潭医院共建奥体创伤医院，市第二医院与积水潭医院、第四医院与同仁医院共建专科，下花园医院与北京中医院共建医联体等事宜。力促北京阜外医院在涿鹿建设亚太第一专科医院、北京佑安医院在经开区建设京张国际肿瘤康复医院项目取得实质性进展。加快推动京张产业共建。加强与北京市经信委合作，引进一批重大产业项目，加快推进中关村昌平园怀来分园、福田汽车产业城、怀来航空航天基地等一批重大合作项目的建设。以打造京张文化体育旅游产业带为载体，引进北京知名公司，促进两地体育、文化、旅游产业融合发展。与北京投资促进局联合举办招商推介活动，重点引进一批新项目、大项目。积极增进与北京的科技合作。与北京科委签订科技奥运合作协议，推进张家口创新创业孵化中心、涿鹿国家级科技孵化器建设，引进一批科技型企业。把怀来高新技术开发区作为与中关村对接的先行区和示范区，制定优惠政策，探索总结经验，不断深化与北京在人才引进和科技成果转化方面的合作。与中国农科院和中国农大共同实施一批现代农业科技成果转化项目。

3.勇于创新，加快可再生能源的转化应用。认真落实《河北省张家口市可再生能源示范区发展规划》，全面启动三年行动计划，在科学化、规模化、规范化、多形式开发建设发电站的基础上，坚持本地消纳和合理外送相结合，重点在可再生能源的应用方面求突破。积极向国家争取政策。加强与国家部委的对接，积极争取在指标、电价、财税、输电等方面给予我市特殊政策。尽快实施一批重点项目。启动张北1000千伏特高压输变电工程的前期工作，推动我市到北京柔性直流电网工程，大力提升可再生能源电力的外送能力。推动沽源风电制氢、张北大数据中心电力消纳等项目，形成可再生能源综合利用产业链。加快实施建工学院风电供暖等一批示范工程，年内全市新能源电力供暖面积达到200万平方米。大力开展应用综合创新。继续巩固与中国科学院的合作，组织成立高水平的专家指导团队，充分利用国家电网节能公司先进的应用技术、设备和新型的商业模式，借助新奥集团等能源互联网平台，科学推进可再生能源高效、低成本综合应用，逐步推行“以电代煤、以电代油”工程，引领和推动我市尽早实现可再生能源替代传统化石能源的战略性调整。启动实施分布式发电、输电、利用一体化项目。同时，谋划实施生物天然气、规模化沼气和生物质燃料替代燃煤等农业可再生能源循环利用工程。

(二)坚定不移促进转型，推动产业结构调整取得实效。抓住供给侧结构性改革政策的窗口机遇期，大力提升传统产业，加快发展新兴产业，积极培育新供给和新产能，提高发展质量和效益。

1.抓项目带升级，大力提高产业发展层次。加大传统产业改造力度。落实工业转型发展八项措施，设立企业转型升级专项基金，抓好50项重大工业项目。构建本地企业产品配套、优势互补、相互支撑的机制和平台，促进企业加强合作，扩大市场，降低成本，提高效益。对中煤机、宣钢、宣工、探机、盛华等重点企业，以转型升级为重点，促使企业推行新技术，开发新产品，提高生产经营效益。对一些长期亏损、债台高筑、高耗低效的企业，通过兼并重组、债务重组或者跨区域、跨领域整合，逐步“激活”。对少数“无药可治”的“僵尸企业”，进行破产清算，实现市场出清。引导传统零售业转变销售模式，尽快向体验式消费转型，向线下选购、线上支付的智能化店铺转型，大力发展乡村物流配送服务，满足不同层次消费者的需求。加快发展战略性新兴产业。以“高端、智能、绿色”为引领，以产业园区为平台，以“基金+项目+创新”为主要模式，助力“四大两新一高”主导产业发展壮大。抓好沃尔沃汽车制造基地建设，开展智能工厂和数字化车间建设试点，确保年内实现缸体缸盖量产出口、整车下线，同时加大招商引资力度，大力引进一批国内外知名汽车零部件企业到园区落户，延长汽车产业链。加快张北云计算产业园建设，确保阿里数据港上半年投入使用，年内争取数据中心实检测试、新能源大数据、移动互联网等项目落地，推动大数据产业加快发展。提高“张江分园” 项目建设速度，特别要引进更多的高新技术项目，尽早见到实效。推进西山生命科学园、应急装备研发制造基地及新能源装备制造等重大项目，壮大高端制造产业。积极争取创建国家级园区和国家新型工业化产业示范基地。大力发展现代服务业。围绕冬奥会、协同发展和示范区建设，拓展服务业新领域，培育新型业态。构建 “大旅游”格局，推动旅游与文化、体育、美丽乡村、造林绿化等相融合，重点打造草原山地游、民俗文化游、冰雪游等精品线路，唱响避暑、避雾、避霾品牌，推进和完善草原天路、崇礼滑雪、桑干河、蔚县暖泉古镇、沽源草原天际线、泥河湾公园等一批重点项目。突出抓好环境卫生整治、服务质量提升、要素配套建设等工作。引进专业化、实力强的大公司开发文化旅游资源，全面提升旅游发展水平。构建京张一体化的旅游大市场，加快开发多元化、小众化、高端化的旅游产品，推动旅游业发展从观光为主向休闲度假、健康养生、体验玩乐为主转变。大力推动电子商务进农村、进社区示范工程，引导扶持电商平台建设，培强壮大大好河山商汇网、怀来天元玻璃网、沽源旅游网、阳原国际裘皮在线商城等电子商务龙头平台。全力争取北京潘家园旧货市场迁入我市，积极跑办怀来免税直销政策落地。推动京张奥物流基地等现代化、专业化的物流园区建设，促进物流信息化、标准化、集约化。持续推进现代农业发展。提升做强畜牧、蔬菜、果品、马铃薯四大产业，培育壮大张杂谷、草业、种苗、花卉四大特色产业。实施旗帜乳业二期、中粮百万头生猪养殖、雪川马铃薯深加工、怀来葡萄酒庄等产业化龙头项目，推动一二三产业融合发展。加快山区综合开发，继续抓好现代农业示范园区建设，加大土地流转力度，扶持种养大户、家庭农场等新型农业主体，打造“大农业、大牧业”发展格局。积极发展壮大林下经济、林产品加工、森林旅游等林业产业，实现林业生态效益和经济效益的双赢。

2.抓治理保升级，持续提升区域生态环境质量。启动实施“多种树、兴水利、快转型”三个专项规划，系统性、全方位、精准化地推进生态治理，逐步形成绿色生产方式和发展方式。以更高的标准造林绿化。积极创新造林、管护、考核、融资“四项机制”，实施好奥运、村庄绿化等五大“增绿工程”。全年完成林业生态建设任务225万亩，森林覆盖率净增2个百分点以上。高标准完成赛事核心区、重点通道、迎宾廊道及第一可视面4万亩的绿化任务，打造标志性的精品工程。以更严的措施治理污染。编制并实施《全市清洁空气行动计划(2016-2022)》。年内，主城区10蒸吨及以下燃煤锅炉全部淘汰，城郊及县城淘汰50%，对保留的燃煤锅炉实施污染防治设施升级改造，对202台工业锅炉实施脱硫脱硝和除尘深度治理。对371家煤炭经营企业实施全封闭改造和关停取缔。通过市场化和法制化手段，关停对生态环境造成严重破坏的露天开采矿山。深入整治建筑施工扬尘和餐饮油烟污染。在主城区及怀来县禁限放烟花爆竹。下大力推进秸秆综合利用，严厉打击秸秆焚烧行为。以更大的力度兴水节水。开工建设乌拉哈达水利工程，争取云州水库调水二期工程批复立项，支持农民多种形式兴建各种蓄水设施。继续推进坝上高效节水灌溉推广和坝下大中型灌区节水改造，年内坝上地区新增高效节水灌溉面积9.39万亩，覆盖面达到86%。加强湿地保护，重点实施沽源闪电河、康保康巴淖尔、尚义察汗淖尔、怀来官厅水库4个国家湿地公园保护与恢复项目，年内力争新增3处国家级湿地公园、1处省级湿地公园。

3.抓创新促升级，激发经济发展新活力。大力推动体制机制创新。深化“放管服”改革，实行“断腕”式的简政放权，推动固定资产投资网上并联审批，加快行业协会商会与行政机关脱钩，建立事权与支出责任相适应的财政体制。制定完善人才使用政策和配套措施，引进留住外来人才、培养用好本地人才，推进人才战略的深入实施。进一步落实和优化“百名硕博”引进政策，加大对到我市工作的留学回国人才、博士后、高层次创业人才的资助力度。年内引进国家千人计划等高层次人才团队5个以上。大力推动科技创新。建立政府引导、企业主体、社会参与的科技创新机制，引进建设一批科技创新平台和国家级企业技术中心、工程实验室，组建张家口可再生能源技术研究院，重点支持杂交谷子国家工程技术中心、能源采掘装备研发实验中心、拉曼光学工程技术研究中心等重点实验室建设。强化企业创新主体地位，制定并实施高新技术企业三年倍增和科技型中小企业成长计划，设立科技成果转化基金，年底高新技术和科技型中小企业分别达到45家和900家以上。大力推动大众创业万众创新。抓好创新驿站、众创空间、科技企业孵化器、知识产权代理机构等科技服务平台的建设，建成省级众创空间2家、市级众创空间6 家。激励高校毕业生和高端人才来张创业，推进高层次人才创业园和大学生创业园建设，年内创业园、孵化基地达到20家以上。落实国家和省关于“双创”的各项政策措施，扶持小微企业逐步壮大。大力推动金融创新。抓紧落实我市与各金融机构的合作协议，不断增强融资能力。积极争取国家和省创业投资引导基金和科技成果转化基金，设立我市中小微企业发展基金，引导中小微企业和初创企业发展壮大。大力发展股权投资，争取中信投资、华融渝富等一批国内知名、实力雄厚的股权投资基金尽快落地。建立保险费补贴及风险补偿金，解决小微企业融资难题。推动北大青鸟、巡天种业等在深交所挂牌，年内每个县区至少新增一家上市公司。以市商业银行为主体组建金融控股公司。全力争取将特色农作物纳入政策性农业保险险种。

(三)对标加压提高水平，促进社会各层面和谐发展。大力提高各级政府及部门的行政效率，下大力改善城乡社会面貌，让广大人民群众享受更多的发展成果。

1.统筹兼顾，大力提高城乡社会发展水平。补短板、破瓶颈、提形象、增实力，统筹推进城乡社会发展。建设清洁、秩序、便利、文明的中心城区。按照城市新定位，与行政区划调整相协调，高标准启动新一轮城市总体规划编制，推动“多规合一”，确保控制性详规的公开性和强制性。从严控制浅山区开发，保护城市生态系统。大力发展城市经济，统筹搞好城市二三产业布局，在推动现代工业向产业园区集聚发展的同时，加快第三产业优化升级，积极壮大金融商务、休闲娱乐、信息网络、文化创意等现代服务业，保持房地产的适度开发和健康发展，重点把经开区打造成为全市先进要素最密集、城市经济最繁荣的新城区。开展城市地下管网普查，建立地下管网综合管理信息系统，积极争取列入国家海绵城市和地下综合管廊建设试点城市。推进“城中村”、棚户区改造，利用3年时间基本完成改造任务，今年棚户区改造1.4万套、90万平方米。启动国家卫生城市创建工作，实施主城区景观提升工程，分类制定城市容貌建设、改善的标准和规范，对建筑立面、广告牌匾、夜景照明、马路市场等实施综合治理，打造城市亮点，提升总体风貌。提高城市管理水平，完成管理体制调整，实行综合执法、重心下移。大力推行政府购买服务、市场化运作的模式，以城市环卫市场化为样板，逐步将供水、供暖、污水、停车、城市广告等城市管理职能和公共服务推向市场。强力整治城乡结合部、背街小巷、临街底商门前等部位的脏乱差顽疾，认真解决小区物业、占道经营、狗患反弹等群众长期关注的问题，确保年内有根本性改观。推进“智慧交通”，科学合理设置交通信号灯和道路行驶指示标志，最大限度利用好道路资源，有效解决交通拥堵、秩序混乱、通行不畅等问题。加快县域建设和发展。大力构建县域现代产业体系，每个县区重点发展2-3个特色鲜明的支柱产业，以重点企业为龙头，打造上下游配套产业集聚的专业园区。实施县城建设三年攻坚行动，拉开框架、完善功能、增加绿量、美化环境，所有县城集中供水、集中供热或清洁能源供热、污水和垃圾无害化处理均要实现全覆盖。认真组织好县城环境卫生、交通秩序、广告牌匾整治三大行动计划，今年必须先让县城干净起来、规范起来。年内力争全市城镇化率提高1.2个百分点，达到52.5%。涿鹿、赤城争创国家园林县城，尚义、阳原、康保创建省级园林县城，怀来达到省级生态园林县城标准。推进脱贫攻坚和美丽乡村建设。细化“六个一批”的具体工作措施，将每一条措施落实到每一个贫困户。重点发展种、养、加特色农业，积极培育旅游、光伏、生态扶贫等新业态，确保贫困农民稳定增收。促进劳动力外出转移就业与支持贫困乡村农民工返乡创业相结合，每个县至少建立一个返乡创业园。认真落实国家和省各项扶持政策，加大各级政府和社会各阶层的帮扶力度，确保贫困群众摘下穷帽子，过上好日子，年内实现25万贫困人口脱贫。将美丽乡村建设与脱贫攻坚统筹推进，尊重农民意愿，强化市场运作，组建筹资平台，抓好1个省级重点片区、4个市级重点片区、10 个中心村、11个旅游村、350个重点村的建设任务。提高广大群众文明素质。围绕筹办冬奥会，以创建全国文明城市为载体，从今年开始全面实施居民素质提升工程，通过宣传、教育、惩戒等形式，深入开展“迎奥运、讲文明、树新风”和“爱我家园、告别陋习”等主题活动，加强法治宣传教育，增强全民法治观念，全面推进奥运城市共治、共建、共享，下大力塑造“文明、友善、大气、包容”的城市形象。

2.以人为本，大力提高社会治理和民生保障水平。继续实施一批普惠民生工程，加强社会综合治理，保持社会大局和谐稳定。认真办好十件民生实事。教育惠民，建院旧校区改建四中、经开区中学综合楼、建院小学、经开区第三小学主体完工，力争投入使用。盛华小学、赐儿山街小学开工建设。全市范围内实现小学六周岁入学。免除公办高中、中职、普通高校贫困家庭学生的学费、住宿费和教科书费。加快张家口学院、河北建筑工程学院研究生院筹建工作。扩大就业，实现城镇新增就业4.5万人，农村劳动力转移就业3.5万人，高校毕业生就业率达到85%以上。社会保障，非公有制企业、农民工、灵活就业人员参加社会保险净增参保人数达到2万人。将农民工、个体工商户、自由职业者全部纳入住房公积金缴存对象。将农村低保标准从2250元/年提高到2900元/年，实现低保线与扶贫线“两线合一”。医疗救治，启动市级公立医院综合改革试点工作。全面实施大病保险。完成市第四医院新址搬迁。养老服务，新增加民办养老机构床位1000张以上，建设71所农村互助养老院。文化事业，启动长城和桥西区堡子里保护规划编制工作。组织系列文化惠民和地方戏曲展演活动。城市重点部位实现公益广告全覆盖。体育活动，市奥体中心完成征地拆迁、规划设计、前期手续，力争开工建设。完成五一广场在盛华地块还建体育场馆工程的前期手续，并争取开工。建成社区健身工程24个、主城区笼式足球场2个，开展多种类、多形式的体育活动。集中供热，中心城区新增集中供热面积370万平方米，集中供热普及率达到95%。 2016年采暖期，实施按热计量收费面积达到300万平方米以上。城市交通，完成北绕城高速北环与东、西环连接。完成敬业街、长治东街、广场东街等建设任务，实施长城西大街西延线、容辰大街、北关大街、通顺路4条道路及4条便道的新建工程。城区绿化，启动实施八角台、东山浅山区绿道绿廊一期工程，以及10 个公园建设工程。打造平安和谐城市。继续开展国家食品安全试点城市和省级食品药品安全县区创建工作，推进食品安全追溯体系、食品生产标杆企业、种养示范基地等引领工程。深入开展国家安全发展示范城市创建工作，强化重点行业领域专项整治，坚决遏制重特大安全事故发生。强化公共安全保障，完善和落实重大决策社会稳定风险评估机制。创新社会治理，鼓励和支持社会组织参与社会治理公共服务，推动政府治理与社会自我调节、居民自治良性互动。大力推进信访法制建设，畅通民意诉求表达渠道，着力打造联系群众、服务群众的新平台。依法严厉打击各类违法犯罪，切实解决好群众反映强烈的社会治安问题。

3.务实为民，大力提高各级政府治理水平。坚持担当务实，打造实干政府。面对新形势和新要求，解放思想，敢于担当，勇于攻克政策、土地、资金、规划等方面的难关，在影响发展的关键环节取得突破。对各项重点工作严格执行贯穿到底的责任落实机制，定标准、定时限、定责任，强化督查问责，严格要账兑现。把夙兴夜寐、激情工作作为常态，用真抓实干、埋头苦干赢得更大的业绩。坚持转变职能，打造高效政府。坚决取消一切束缚发展的行政权力，坚决砍掉一切多余的手续程序，坚决向不作为、慢作为、乱作为开刀，进一步优化审批流程，努力创优营商环境。认真践行“三严三实”，在全市政府系统深入开展办事效率和服务质量“双提升”活动，切实解决办事不严格、低标准、低效率、混日子的问题，大幅提高政府机关工作水平。坚持依法行政，打造法制政府。严格落实《法治政府建设实施纲要》，完善依法行政制度体系，实行政府法律顾问制度，加强重点领域政府立法，推进行政决策科学化、民主化、法治化。严格规范公正文明执法，强化对行政权力的制约和监督，有效化解社会矛盾纠纷，提高政府工作人员法治思维和依法行政能力，加快法治政府建设。自觉接受人大法律监督、政协民主监督和社会广泛监督，认真听取各方面的意见和建议。充分发挥审计监督职能作用，对公共资金、国有资产、国有资源和领导干部履行经济责任情况进行审计全覆盖。坚持廉洁自律，打造干净政府。把纪律和规矩挺在前面，落实廉洁自律的各项制度和规定，抓好政府系统廉政建设，坚决查处腐败问题。厉行勤俭节约，严控一般性支出，全面完成公务用车制度改革，确保把有限的资金用在刀刃上、用在办大事上、用在保障民生上。
